# Digital-Tier Strategy Improves Newborn Screening for Glutaric Aciduria Type 1

**DOI:** 10.3390/ijns10040083

**Published:** 2024-12-21

**Authors:** Elaine Zaunseder, Julian Teinert, Nikolas Boy, Sven F. Garbade, Saskia Haupt, Patrik Feyh, Georg F. Hoffmann, Stefan Kölker, Ulrike Mütze, Vincent Heuveline

**Affiliations:** 1 Engineering Mathematics and Computing Lab (EMCL), Interdisciplinary Center for Scientific Computing (IWR), Heidelberg University, 69120 Heidelberg, Germany; contact@saskiahaupt.de (S.H.);; 2 Data Mining and Uncertainty Quantification (DMQ), Heidelberg Institute for Theoretical Studies (HITS), 69118 Heidelberg, Germany; 3 Division of Pediatric Neurology and Metabolic Medicine, Department of Pediatrics I, Center for Pediatric and Adolescent Medicine, Medical Faculty of Heidelberg, Heidelberg University, 69120 Heidelberg, Germany; julian.teinert@med.uni-heidelberg.de (J.T.); nikolas.boy@med.uni-heidelberg.de (N.B.); sven.garbade@med.uni-heidelberg.de (S.F.G.); patrik.feyh@med.uni-heidelberg.de (P.F.); georg.hoffmann@med.uni-heidelberg.de (G.F.H.); stefan.koelker@med.uni-heidelberg.de (S.K.);

**Keywords:** artificial intelligence, data mining, Glutaric acidemia type 1, machine learning, neonatal screening

## Abstract

Glutaric aciduria type 1 (GA1) is a rare inherited metabolic disease increasingly included in newborn screening (NBS) programs worldwide. Because of the broad biochemical spectrum of individuals with GA1 and the lack of reliable second-tier strategies, NBS for GA1 is still confronted with a high rate of false positives. In this study, we aim to increase the specificity of NBS for GA1 and, hence, to reduce the rate of false positives through machine learning methods. Therefore, we studied NBS profiles from 1,025,953 newborns screened between 2014 and 2023 at the Heidelberg NBS Laboratory, Germany. We identified a significant sex difference, resulting in twice as many false-positives male than female newborns. Moreover, the proposed digital-tier strategy based on logistic regression analysis, ridge regression, and support vector machine reduced the false-positive rate by over 90% compared to regular NBS while identifying all confirmed individuals with GA1 correctly. An in-depth analysis of the profiles revealed that in particular false-positive results with high associated follow-up costs could be reduced significantly. In conclusion, understanding the origin of false-positive NBS and implementing a digital-tier strategy to enhance the specificity of GA1 testing may significantly reduce the burden on newborns and their families from false-positive NBS results.

## 1. Introduction

Worldwide, newborn screening (NBS) programs aim to identify newborns with treatable severe rare diseases at an early stage, ideally pre-symptomatically, to enable an early start of treatment. Therefore, blood samples from newborns are collected on the first days of life (i.e., in Germany at 36–72 h of life) and sent to an NBS center for analysis. Hence, NBS is considered a highly effective public health program for secondary prevention [1,2]. Glutaric aciduria type 1 (GA1; OMIM: #231670) is a rare autosomal recessive inherited metabolic disease and part of NBS, with an estimated birth prevalence of 1:135,000 newborns in Germany [1]; however, it is found at a significantly higher prevalence in known high-risk populations [3,4,5]. The disease is characterized by an accumulation of glutaric acid, 3-hydroxyglutaric acid, and glutarylcarnitine (Glut) [6]. Untreated, 80% to 90% of patients with GA1 experience a metabolic decompensation and neurological complications due to striatal injury, particularly a complex movement disorder with predominant dystonia [6,7,8]. Despite its severe clinical manifestation, GA1 is considered a treatable disease in screened populations, since early diagnosis and the start of treatment improve the outcome [6,9,10,11,12]. Based on their amount of urinary excretion of glutaric acid, high (>100 mmol/molCrea) and low excretors (≤100 mmol/molCrea) are distinguished [13]. Importantly, low excretors have a similar risk for movement disorders, but often show only slight elevation or even normal concentrations of Glut, the primary NBS parameter. This has led to the need for relatively low cut-off values for Glut in NBS to avoid false-negative results [2,14,15]. This allowed a sensitivity of 95.6% for GA1 in Germany during 1999–2016 [2] at the cost of a high number of false positives, lower specificity and positive predictive value (PPV). Moreover, other factors influencing Glut levels and leading to false positive NBS results have been reported, e.g., renal insufficiency of the newborn [16,17]. Due to the relative high number of false positives, confirmatory diagnostics for suspected GA1 cases in NBS programs are frequently employed and associated with follow-up costs. They involve reanalysis of acylcarnitines, analysis of urinary 3-OH-GA concentration, and in some cases genetic and/or enzymatic testing to further support or rule out the diagnosis [12]. In recent decades, especially second or multiple tier testing was established to improve specificity in NBS and to reduce the number of false-positive NBS results, while maintaining 100% sensitivity, and minimize the harm false-positives cause to newborns and their families [18,19,20]. However, biochemical second tier methods for GA1 are not established and, hence, new methods need to be developed.

Recently, there has been a notable rise in the application of data-driven methodologies specifically machine learning (ML) and deep learning aimed at refining classification tasks within medical data sets and NBS contexts [21]. Numerous studies have demonstrated that these methods have the potential to enhance classification accuracy by minimizing false-positive rates and unveiling previously unidentified metabolic patterns within data sets by making use of the information of all metabolite concentrations screened for in NBS [22,23]. Among the previously applied ML methods, logistic regression (LR) and support vector machines (SVMs) showed good performance for NBS classification in single and comparative studies [21]. Moreover, more complex methods such as Feed Forward Neural Networks (FFNN) [24], boosting methods [22], and Random Forest (RF) [23] showed good results in reducing specificity, but did not always maintain 100% sensitivity. Furthermore, the efficacy of a digital-tier strategy, which acts as a second screening step after regular NBS, was previously demonstrated for isovaleric aciduria reducing the high number of false positives by about 70% through a combination of regular NBS with ML classification [25].

This study aims to improve the understanding of false-positive NBS results in regular NBS for GA1, to develop a full data and digital-tier strategy for NBS for GA1 based on ML classification methods, and, finally, to increase the specificity of NBS for GA1 and reduce the burden on falsely suspected newborns and their families.

## 2. Materials and Methods

### 2.1. NBS Data Set-Composition, Extraction and Data Cleaning

Heidelberg University Hospital’s (UKHD) NBS laboratory screens about 20% of newborns in Germany; approximately 140,000 newborns annually. The set of NBS variables was anonymized, and data extraction and evaluation were performed in accordance with the European general data protection regulation (GDPR). Thus, the approval of the local ethics committee was not necessary.

The data set for NBS encompasses a total of 61 variables, including 52 metabolite concentrations alongside birth weight, sex, gestational age, birth year, age at blood sample collection, sample arrival time, indicators specifying suspected and subsequently confirmed diagnoses, and comments on clinically relevant information (Supplementary Table S1A). To ensure the validity of the data set, it was meticulously narrowed down. It includes only the initial NBS results of newborns born at a minimum gestational age of 32 weeks, aged at least 36 h at the time of sampling, and possessing unremarkable NBS reports, herein referred to as ‘normal’. Additionally, all profiles of newborns flagged with suspected GA1, whether confirmed or later excluded as false positives, were isolated. The NBS data set comprises profiles from a total of 1,055,885 newborns. This includes 604 cases flagged as suspected GA1, and nine with confirmed GA1, spanning births between 2014 and 2021. In this period, no false negative GA1 case was reported. For Heidelberg GA1 screening, this leads to a sensitivity of 100%, a specificity of 99.94%, a false-positive rate of 0.06%, and a PPV of 1.5% between 2014 and 2021. Data cleaning procedures were executed on the extracted data set to uphold high data quality standards. Supplementary Figure S1 shows the data extraction and data cleaning steps performed on the data set. To achieve this, specific ranges were defined to exclude data sets containing implausible values: birth weight: 1000–6000 g; gestational age: 32–42 weeks; age at sampling: 36–120 h; age at sample arrival: 0–20 days; and metabolite concentrations: 0–50,000 μmol/L. Moreover, categorical values underwent conversion into numerical representations. In addition, three metabolite variables, namely glutamine, succinylacetone, and immune reactive trypsin, were excluded from the data set due to a substantial number of missing values, attributed to their intermittent measurement within the designated time frame.

Finally, the total data set for analysis (hereafter “full data set”) contained 1,025,953 NBS profiles (including 494 cases with the suspected diagnosis GA1, hereafter “suspected diagnosis data set”); see Supplementary Figure S1. The suspected diagnosis data set included nine subsequently confirmed GA1 cases, with six low excretors, three high excretors, and 485 confirmed false positives. For newborns with suspected GA1, comments indicating symptoms or treatments of newborns (e.g., parental nutrition, medication, transfusion, severe comorbidities, etc.) on the initial NBS card were retrieved. Moreover, results of quantitative analysis of urinary 3-OH-GA with stable isotope (normal, elevated) and results of further testing to disprove or confirm the suspected diagnosis of GA1 were retrieved in cases where the information was returned to the UKHD NBS laboratory. If no information on further testing was available, profiles were marked as lost to follow up.

Additionally, an independent test data set from the NBS laboratory in Heidelberg was extracted after the ML algorithms’ initial training and validation. The test data comprised screening profiles from January 2022 to October 2023, and consisted of 257,414 NBS profiles. The test data set was curated based on the same data cleaning and exclusion criteria as the original GA1 data set.

### 2.2. Data Analysis Methods

Data analysis methods aim to learn information and patterns from large amounts of data which support an ML-based classification method. In a binary classification problem, the data points $x_{i}\in R^{m}$ belong to one of two classes, $x_{i}\in C_{0}$ or $x_{i}\in C_{1}$.

#### ANOVA

Analysis of Variance (ANOVA) [26] is a statistical method that assumes the underlying data $X\in R^{m\times n}$ with *m* features and *n* data points to be normally distributed. ANOVA tests whether the means $\mu_{0},\mu_{1}$ of different classes $C_{0},C_{1}$ are significantly different. This method is evaluated with a predefined *p*-value. The null hypothesis $H_{0}$ assumes that the means of the two classes are equal,

(1) $$H_{0}:\mu_{0}=\mu_{1}.$$

The alternative hypothesis $H_{A}$ assumes that the means are different,

$$\begin{array}{c} H_{A}:\mu_{0}\neq \mu_{1}. \end{array}$$

By this, the ANOVA can be applied to test whether the mean values of a feature differ between classes.

### 2.3. Machine Learning Classification Methods

ML classification algorithms attempt to learn a pattern for a classification task based on a labeled data set by updating internal model parameters. For the ML evaluation, the best-performing algorithms in comparative studies on ML-based NBS were applied [21,25].

#### 2.3.1. Logistic Regression

Logistic regression (LR) stands as a discriminating approach, focusing on modeling the posterior probability distribution P(Y|X) of the target variable *Y* given the features *X*. This method draws from linear regression principles centered on capturing linear relationships within the data by identifying the most suitable linear model,

(2) $$\hat{y}=\beta_{0}+\beta_{1}\cdot x_{1}+\beta_{2}\cdot x_{2}+\ldots +\beta_{m}\cdot x_{m},$$

through adjusting the regression coefficients $\beta_{0},...,\beta_{m}$. Since LR is designed to estimate the probability of a data point belonging to a particular class, it employs a probability measure of class membership of the feature vector,

$$P\left(y=1|X=x_{i}\right)=\frac{1}{1+e^{-(x_{i}^{T}\beta )}}.$$

During training, the regression coefficients are fitted using a maximum log-likelihood method to maximize the probability of obtaining the observed results with the fitted coefficients [24,27,28].

#### 2.3.2. Ridge Logistic Regression

Ridge logistic regression (RR) enhances the LR model by imposing penalties on the complexity of the resulting model. Therefore, an additional regularization parameter λ>0 is added to the LR function, and an additional ${\lambda ∥\beta ∥}^{2}$ is added to the log-likelihood [29,30]. In the RR optimization, coefficients are constrained by the square of the Euclidean norm of the coefficients. Hence, the regularized log-likelihood is

(3) $$l_{r}\left(\beta \right)=l\left(\beta \right)-\frac{\lambda }{2}\sum_{k=1}^{m}\beta_{k}^{2},$$

where β are the regression coefficients and the penalty parameter λ regulates the degree of shrinkage towards zero [31].

#### 2.3.3. Support Vector Machines

Support vector machines (SVMs) attempt to find a separating hyperplane between two classes by transforming the features $x_{i}$ of a data point $x\in R^{m}$ into a higher dimensional space [24,28]. A linear hyperplane can be written as

(4) $$w^{T}x+b=0,$$

where $w\in R^{m}$ is the orthogonal vector to the hyperplane, and $b\in R^{m}$ is the distance of the hyperplane from the origin. A margin can be defined for the data point $x\in R^{m}$. A true positive *x* has a margin of (w·x+b)>0, and a true negative *x* has a margin (w·x+b)<0. The data points *x* nearest to the decision boundary are called support vectors. An SVM determines the decision boundary as a linear combination of support vectors. In the case of a hard-margin linear SVM classifier, maximizing the margin entails solving a quadratic, constrained optimization problem to determine the optimal parameters *w* and *b*. However, data points from distinct classes cannot be effectively separated in many scenarios by a linear decision boundary. Therefore, kernel functions $K\left(\cdot ,\cdot \right):R^{n}\times R^{n}\to R$ are applied for non-linear SVM. These transform the input data into a high-dimensional feature space where the data are linearly separable.

### 2.4. Experimental Setup

For all evaluations, we applied the programming language Python (Python Software Foundation; Python Language Reference, version 3.9.2, available at http://www.python.org (accessed on 12 March 2023) and the Python libraries scikit-learn [32] (version 1.0.2) and scipy [33] (version 1.10.1). Each ML method was evaluated on the full data set and in a digital-tier strategy. The digital-tier strategy combines regular NBS with the subsequent application of ML methods to the suspected diagnosis data sets. To address the data imbalance, the optimal class weight parameter $w_{0},w_{1}$ for each classification method was identified to penalize misclassification of true positives more heavily in the cost function during optimization [32]. Therefore, the majority class weight parameter $w_{0}$ is set to 1, and a grid search to determine the optimal value for the minority class weight parameter $w_{1}$ is applied.

### 2.5. Validation

The suspected diagnosis and the full data sets were randomly split into 80% training and 20% validation sets for evaluation. The classification performance on both data sets was evaluated with the confusion matrix *C*,

$$C=\left[\begin{array}{cc} TN & FP\\ FN & TP \end{array}\right],$$

with true negatives (TN), false positives (FP), false negatives (FN), and true positives (TP). These results were then validated with ten repeats of 5-fold cross-validation on the two objectives, maintaining 100% sensitivity $S_{n}$ and maximizing specificity $S_{p}$,

$$S_{n}=\frac{\mathrm{TP}}{\mathrm{TP}+\mathrm{FN}},\;\mathrm{and}\;S_{p}=\frac{\mathrm{TN}}{\mathrm{TN}+\mathrm{FP}}.$$

For the digital-tier strategy, the specificity is reported as combined specificity using ML as an additional step to traditional NBS for comparability with the results on the full data set. Furthermore, the ML algorithms were tested on an independent test data set which was extracted after the algorithms’ initial training and validation.

## 3. Results

### 3.1. Data Analysis

**Figure 1:**
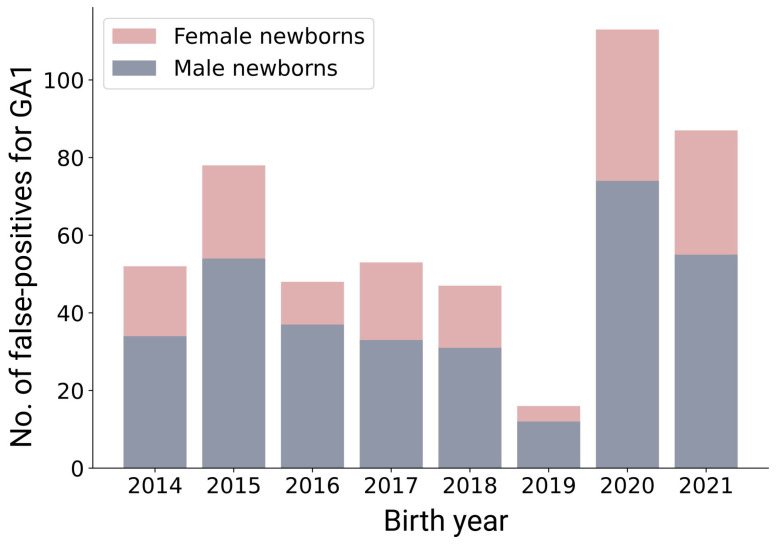
False-positive newborn screening results for GA1. Sex-specific differences in false-positive newborn screening results for GA1 from 2014 to 2021.

The data analysis revealed a sex imbalance in the suspected diagnosis data set (Figure 1). The false positives were divided into 326 (67%) males and 159 (33%) females, whereas GA1 was similarly prevalent in male (*n* = 4) and female (*n* = 5) newborns. This finding was stable across the birth years 2014 to 2021, with a larger number of false-positive males than females in every year investigated, Figure 1. This imbalance could be caused by differences in the distribution of the Glut value in male and female newborns (ANOVA p<0.0001), as the mean Glut value of newborns without GA1 is 0.16±0.058 in males and 0.15±0.055 in females. An evaluation of false-positive NBS profiles on the test data set from 2022 to 2023 also revealed sex-specific differences, as the data set consisted of 135 (57%) males and 100 (43%) females.

**Figure 2:**
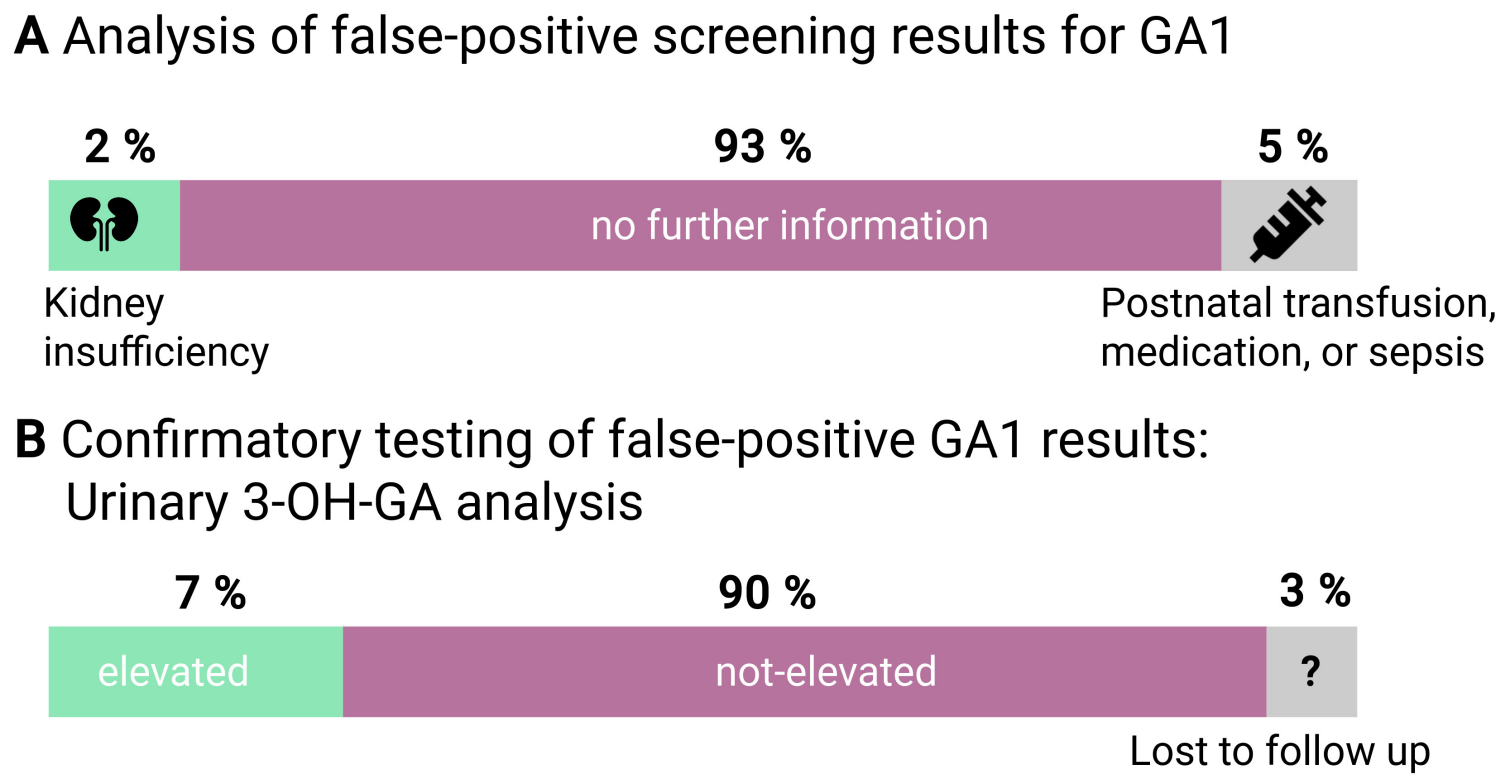
Further analysis of 485 false-positive screening results in GA1. (**A**) Reports of kidney insufficiency (*n* = 10) and transfusion, medication, or sepsis (*n* = 23) in false-positive newborn screening results for GA1. (**B**) Evaluation of urinary 3-OH-GA analysis in false-positive newborn screening results for GA1 including 34 (7%) newborns with elevated 3-OH-GA.

While for 93% (*n* = 452) of newborns with false-positive screening results, no symptoms or treatments were indicated at NBS sampling, for 2% (*n* = 10) there was kidney insufficiency, and for 5% (*n* = 23), there were other major abnormalities potentially interfering with NBS results reported, such as postnatal transfusion, medication or sepsis (Figure 2A). In the false-positive data set, GA1 was excluded by quantitative analysis of urinary 3-OH-GA in 90% of the profiles (*n* = 435). In 7% (*n* = 34) of all suspected cases who were finally excluded as false positives, urinary analysis revealed elevated 3-OH-GA levels, prompting further evaluations such as genetic testing, enzymatic testing, or both to rule out the diagnosis. For 3% (*n* = 16), no follow-up data on urinary 3-OH-GA concentration was available (Figure 2B). For three patients in the false-positive data set, the UKHD NBS laboratory received feedback on the presence of a heterozygous pathogenic *GCDH* variant after genetic testing. All newborns heterozygous for GCHD showed elevated urinary 3-OH-GA, whereas none of the newborns with renal insufficiency had elevated 3-OH-GA levels (Supplementary Table S2).

Furthermore, we applied ANOVAs to compare the mean and standard deviation of measured metabolites in dried blood samples across three distinct groups: normal NBS profiles, false-positive NBS profiles, and newborns diagnosed with GA1 (Supplementary Table S1B).

As expected, the mean (±standard deviation) concentration of Glut was highly elevated in newborns with GA1 (2.698±1.548μmol/L) compared to newborns with suspected, but not confirmed GA1 (0.526±0.106μmol/L, ANOVA p<0.0001) and newborns with normal NBS profiles (0.157±0.057μmol/L, ANOVA p<0.0001); see Supplementary Table S1B. The mean and standard deviation of Glut in GA1 patients was 1.9±1.28μmol/L in the six low excretors, and 4.3±0.2μmol/L in the three high excretors.

Then, we performed ANOVAs with significance levels of 5% (*p*-value <0.05) on the full GA1 data set, comparing the metabolic profiles of patients with GA1 and all other NBS profiles (Table 1A). Furthermore, we compared the metabolic profiles of individuals with confirmed to those of individuals with false-positive NBS results using ANOVA with significance level of 5% (*p*-value <0.05) (Table 1B), and confirmed the known biomarker Glut as a significant feature in both data sets (Table 1). However, further significant features (e.g., homocitrulline (Hci), isovalerylcarnitine (C5)) were present in the full data set. In contrast, other parameters (e.g., decanoylcarnitine (C10), tetradecenoylcarnitine (C14:1)) were identified as significant features with the highest F values by the ANOVA in the suspected diagnosis data set (Table 1). The box plots (Supplementary Figure S3) provide a detailed view of these features for both data sets. Overall, the mean and the median concentrations of most acylcarnitines, e.g., C10, are higher in the false-positive group than in newborns with normal NBS profiles or confirmed GA1 (Supplementary Table S1B and Supplementary Figure S3). The ANOVA was then used as a feature selection method to reduce the dimensionality of the data set for the ML methods.

### 3.2. Machine Learning Results for Full and Suspected Diagnosis Data Set

The digital-tier strategy describes an additional step after the first newborn screening. Here, the newborn screening profiles of all newborns that are suspected positive by the initial newborn screening are classified, in analogy to biochemical second-tier methods, with a machine learning method into ‘normal’ and ‘suspected GA1’ profiles, and by this the number of false positives that need to be further analyzed is reduced. For the evaluation of the machine learning approach on the full data set, as well as on the suspected diagnosis data (digital-tier strategy), we compared the classification results of different classification methods (LR, RR, and SVM), Table 2. Overall, the best methods on both data sets decreased the number of false positives while having no false negatives in the respective training and validation data set (Table 2), which is important since all newborn with GA1 need to be detected correctly. For most algorithms, basing the evaluations on Glut and C10 led to the best results.

On both data sets, the RR method performed worse than LR and SVM in terms of overall false-positive rate reduction. The LR, when only based on the features Glut and C10, presented the best-performing algorithm for reducing false positives while minimizing the number of false negatives for both data sets. LR overall reduced the number of false positives by 93.61% on the full data set, and by 95.05% on the suspected diagnosis data set for training and validation. Despite the LR, RR, and SVM algorithms demonstrating no false negatives on a randomly stratified split training and validation set, the 5-fold cross-validation using stratified splitting revealed that none of the algorithms achieved 100% sensitivity.

Therefore, a grid search with five-fold stratified cross-validation over the applied class weight parameter $w_{0},w_{1}$ was applied to achieve 100% cross-validation sensitivity. The parameter $w_{0}$ was set to $w_{0}=1$, and the optimal parameter $w_{1}$ was searched in the interval $I_{F}=\left[1,50,000\right]$ for the full data set, and in the interval $I_{S}=\left[1,500\right]$ for the suspected diagnosis data set. The best-performing methods in terms of highest mean sensitivity and specificity are presented in Table 2D. Since, on the full data set, no method achieved 100% sensitivity, only the results of the suspected diagnosis data set are shown (Table 2D). All three methods (LR, RR, and SVM) achieved 100% sensitivity in cross-validation, by increasing the class weight $w_{1}$ to values between 180 and 183. However, this weight adaptation also reduced the specificity of all three methods. The LR classification achieved the best results on the training and validation set with 147 false positives, while SVM (164 FP) and RR (235 FP) had higher false-positive rates (Table 2D). Hence, the false positives were reduced by 69.69% with LR, 66.19% with SVM, and 51.55% with RR, compared to traditional NBS, while maintaining 100% sensitivity.

To validate the ML classification results on GA1, we extracted an independent test data set, including data from January 2022 to October 2023, from the NBS laboratory at UKHD. On the test data set, the LR classification method, which was initially trained on the full data set, achieved a reduction in false positives of 93.19% compared to traditional NBS (reduction from 235 to 16 FP results) on the test data set (Table 2B). The LR classification method, which was initially trained on the suspected diagnosis data set, achieved a reduction in false positives of 92.34% compared to traditional NBS (reduction from 235 to 18 FP results) on the test data set (Table 2C). The optimized LR method, classified 115 NBS profiles incorrectly as GA1, translating to a false-positive reduction of 51%. Overall, on the suspected diagnosis data set all ML methods identified patients with GA1 correctly, resulting in 100% sensitivity on the test data set (Table 2).

### 3.3. Machine Learning Results for False-Positive Subgroups

**Figure 3:**
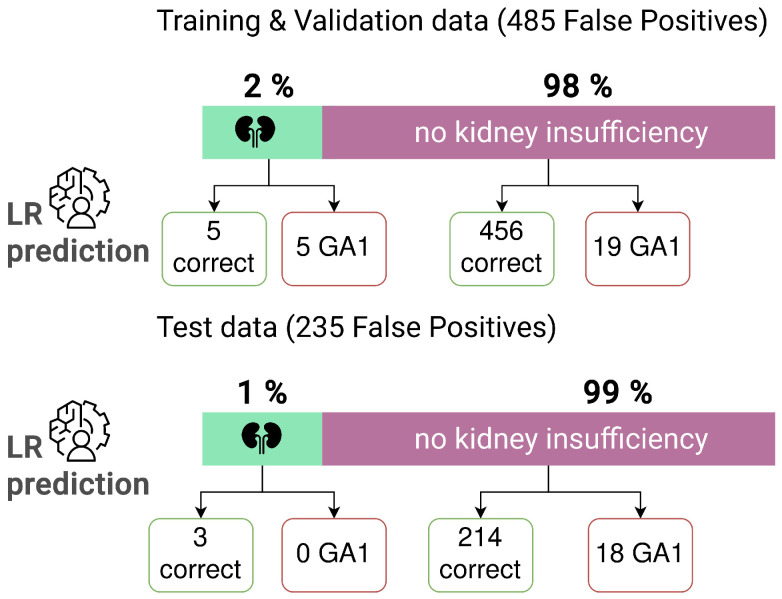
Detailed analysis of classification by LR algorithm of subgroups of false-positive newborn screening results in the training and validation data set (*n* = 485) and the test data set (*n* = 235) for GA1 for newborns with kidney insufficiency (*n* = 10 patients with kidney insufficiency in the training and validation data set, *n* = 3 patients with kidney insufficiency in the test data set).

**Figure 4:**
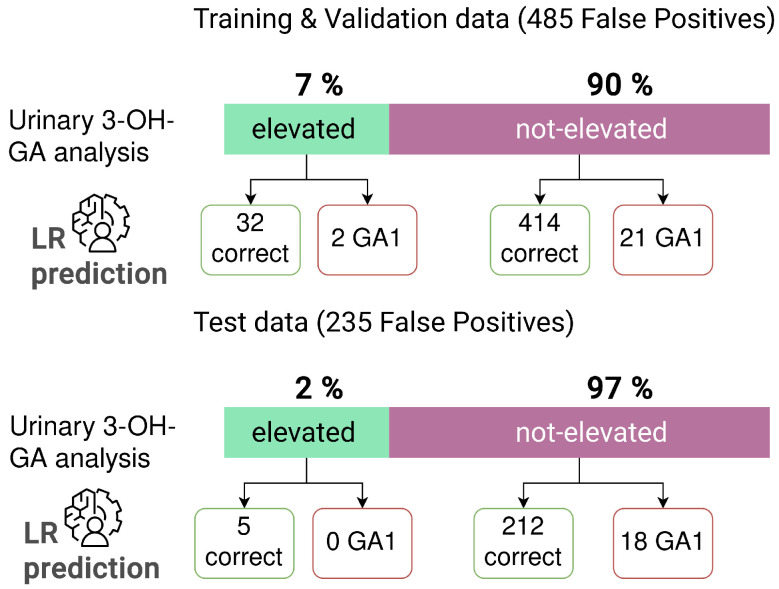
Detailed analysis of classification by LR algorithm of subgroups of false-positive newborn screening results in the training and validation data set (*n* = 485) and the test data set (*n* = 235) for GA1. Evaluation for newborns with elevated urinary 3-OH-GA (*n* = 34) in the training and validation data set and patients with with elevated urinary 3-OH-GA (*n* = 5) in the test data set).

Data analysis identified different subgroups within the false-positive NBS profiles. To evaluate whether the reduction in false positives using ML corresponds to one of these subgroups specifically, we analyzed how these subgroups are divided by the LR classification in the 485 false-positive profiles used for the training and validation, as well as the 235 false-positive profiles used for testing. The digital-tier strategy with LR, which was trained on the suspected diagnosis data set (Table 2C) was applied and NBS profiles of newborns with and without kidney insufficiency, as well as newborns with and without elevated urinary 3-OH-GA were investigated. Analysis of results of 3-OH-GA in urine revealed relatively more (7%) elevated profiles in the original training and validation data set, than in the test data set (2%). Kidney insufficiency was reported in 2% of profiles in the original data and 1% of profiles in the test data set (Figure 3). There was no consistent distinction of newborns with kidney insufficiency or other indicated abnormalities using the LR method: 50% of the profiles with kidney insufficiency in the training and validation data set were classified as GA1, and 50% were classified as normal by the LR method (Figure 3). In the test data set all profiles with kidney insufficiency were predicted to be normal (Figure 3). Similarly, we did not find a complete overlap between the false-positive group identified by the LR prediction and the group with elevated 3-OH-GA, since two of the profiles with elevated 3-OH-GA are falsely classified as GA1, and 21 of the profiles without elevated 3-OH-GA are falsely classified as GA1 (Figure 4). In the test data set, all falsely classified newborns with GA1 are profiles without elevated 3-OH-GA levels (Figure 4).

## 4. Discussion

NBS is a highly successful instrument of secondary disease prevention. However, NBS for rare diseases such as GA1 faces a number of challenges, including false-positive screening results [34]. In this study, an in-depth analysis of false-positive NBS profiles for GA1 was performed, and a digital-tier strategy in which an ML classification method is applied as a second step after regular NBS was developed to decrease the number of false positives.

In the NBS training and validation data set, a false-positive rate of 0.047% (1,026,447 NBS profiles and 485 FP) was observed, which is higher than previously reported rates for other metabolic NBS target diseases [35], highlighting the necessity for improvement. Further investigation of all 720 false-positive screening results (including training/validation and test data set) revealed a sex-specific imbalance. This imbalance appears to stem from differences in the distribution of Glut values between male and female newborns. Future studies are needed to explore the possibilities of sex-specific differences in GA1 screening. Sex-specific ML models for example could be developed to improve classification accuracy. Nonetheless, the low number of confirmed GA1 cases among male and female patients presents a significant challenge for validating these models [36,37].

A comprehensive assessment of the false-positive screening profiles revealed that in the majority of cases, diagnosis of GA1 was ruled out following the analysis of 3-OH-GA in urine samples. However, in accordance with current international guideline recommendations [12], for 34 newborns (7% of the false-positive data set) with elevated 3-OH-GA results, initiation of metabolic treatment and further genetic and/or enzymatic tests was necessary to confirm or exclude the diagnosis, constituting a possible burden for affected families [38]. Additionally, the analysis identified that only 2% of the false-positive screening results were associated with kidney insufficiency, a known cause of false-positive NBS for GA1 results, although previous reports attributed a larger proportion of false-positive screening results in GA1 to kidney insufficiency [16,17]. While it is unclear if this elevation of Glut levels in newborns with kidney insufficiency is caused by disturbances in specific renal transporter systems of glutaric acid and its derivatives, or a general reduction in acylcarnitine excretion, it is important to note that the prevalence of kidney insufficiency in this study might be underestimated, as the condition might not have been diagnosed at the time point of screening and therefore not indicated on NBS cards.

Interestingly, the best-performing ML strategy developed in this study similarly reduced the number of false-positive screening profiles associated with subsequently normal and elevated urinary 3-OH-GA levels, as well as with and without kidney insufficiency. This indicates that the reduction in false-positive results through this strategy is not due to a pattern in the Glut and C10 values used by the algorithm correlating to urinary 3-OH-GA levels or kidney insufficiency. Moreover, it provides evidence that the strategy not only reduces the number of false-positive results in general, but eventually reduces the need for genetic and/or enzymatic testing and associated costs as well, thereby further lessening the impact of false-positive NBS on newborns and their families.

The best-performing ML methods on both the full and suspected diagnosis data sets utilized Glut and C10 as crucial features for classification, confirming well-established knowledge [12]. This consistent importance of the Glut parameter across data sets suggests that reevaluating current cut-off values for GA1 in NBS could benefit future studies. In addition to Glut, the ANOVA on the suspected diagnosis data set identified C10 as a significant feature. Importantly, C10 is known to be elevated in other inherited metabolic disorders, such as multiple acyl-CoA dehydrogenase deficiency, but not in GA1 [39]. In line with this, analysis of acylcarnitine measurements revealed that C10 and other acylcarnitines showed higher mean and median values in the false-positive group compared to those in the unremarkable NBS profiles and newborns with GA1 (see Supplementary Table S1B and Supplementary Figure S1). The high levels of several acylcarnitine measurements could be a potential reason for the high number of false-positive screening results for GA1. These findings suggest that Glut could be complemented by other parameters such as C10 or an overall increase in acylcarnitine profiles to serve as a more effective indicator of potential false-positive screening results for GA1. Furthermore, it has to be considered that the metabolites found to be elevated in NBS might actually represent other compounds that are isobaric and might explain some of the false positives.

ML methods can help to model such complex data relationships, and have recently been shown to improve NBS results [21,22,23,25]. In the training and validation data sets, the LR method demonstrated excellent performance, reducing the number of false positives by 93.61% on the full data set and by 95.05% on the suspected diagnosis data set, simulating a use as a digital-tier after traditional NBS, while correctly identifying all patients with GA1. This high level of performance was maintained on an additionally extracted test data set from newborns born between 2022 and 2023. However, only the LR method with cross-entropy loss function weight $w_{1}$ optimized for cross-validation achieved 100% sensitivity in cross-validation. This method reduced the false-positive rate on the suspected diagnosis data set by nearly 70% in training and validation and by 51% on the test data set (Table 2D). The grid search for optimal class weight parameters was crucial for increasing specificity while maintaining 100% sensitivity, which is essential for NBS to ensure all affected newborns are identified. These results suggest that ML methods can increase specificity, i.e., reducing the number of false positives, in NBS for GA1, especially if used in a digital-tier strategy. Importantly, these methods can be applied automatically within a few minutes at minimal costs. False-positive NBS results incur additional costs and efforts, including the need for physicians to communicate the remarkable results to local hospitals and families, clinical evaluations of the newborns, and sampling for confirmatory diagnostics by pediatricians. These processes also involve expenses for metabolic and genetic analyses. Therefore, ML methods can, by increasing the specificity of the screening, alleviate the burden on infants and their families while enhancing the cost-effectiveness of NBS.

However, due to the small number of true positives (nine affected patients in the training and validation data set), the relevance and significance of the results are challenging to estimate. An unfavorable splitting of the data set in cross-validation could decrease sensitivity. Despite this, both LR methods correctly identified all patients with GA1 in the training, validation, and test data sets (eleven patients with GA1). Moreover, since the LR model was only trained on data from the Heidelberg screening laboratory in Germany, the model may only apply to other NBS laboratories with center-specific data retraining, due to variations in screening procedures, equipment, and materials. Nevertheless, other screening centers could adopt the presented experimental setup and identified features to train an LR model optimized for their specific data sets, thereby improving the false-positive rate for GA1 in their patient populations. For the application of these methods in clinical practice, it must be ensured that the same data ranges such as the exclusion of newborns with a gestational age smaller than 36 weeks, are applied to the new data samples. Analogous to different cut-offs and algorithms for preterm and term newborns in the biochemical NBS. Samples that do not lie within these ranges are then analyzed according to current NBS, and not with a digital-tier.

In the future, a prospective parallel evaluation of the algorithms as a potential clinical decision support system would be beneficial to determine which algorithm yields the best results. Additionally, the findings should be validated on new data sets, including more positive GA1 cases. Moreover, recently post-analytical tools such as the Collaborative Laboratory Integrated Reports (CLIR) Tool, which is based on continuous adjustments of covariates instead of traditional cutoff values are applied in newborn screening [40]. Due to data protection laws and privacy restrictions a comparison between the CLIR tool and the presented method was not possible; however, this could be investigated in future studies. Future studies could also investigate other state-of-the-art ML methods for classification tasks such as Neural Networks [41], XGBoost [42], and LightGBM [43] to improve NBS for GA1. However, for clinicians, an explanation is helpful to estimate the reliability of the system’s decision [44]. Therefore, in the application of more complex methods, their interpretability should also be addressed, such as with the use of explainable artificial intelligence methods, which showed promising results in NBS for isovaleric aciduria [22] and clinical decision support systems [45].

In general, ML methods are expected to be more frequently applied in the clinical context [46]. However, it is not clear how they can be applied in critical areas such as decision support systems. Therefore, it needs to be determined which ethical and legal requirements are needed to apply ML methods [47]. Additionally, addressing patients’ fears of discrimination by the algorithms and securing data protection will be important steps to integrate artificial intelligence into the medical domain. In particular, in personalized treatment and precision medicine, data-based methods could enhance clinical work such as the digital metabolic twins for newborns and infants which predicted known biomarkers and responses to treatment strategies of inherited metabolic diseases [48].

Overall, this study provides evidence that ML methods can be implemented to increase the specificity of NBS for GA1, thereby helping to reduce the possible burden of false-positive screening results on newborns and their families, and offers new perspectives on NBS in the future.

## Figures and Tables

**Table 1:** ANOVA results with presented features that have a *p*-value p<0.05, showing the mean (μ) and standard deviation (σ) of these features in the normal and GA1 NBS profiles. All methods were applied to the full newborn screening (NBS) data set (**A**) and the suspected diagnosis data set (**B**). Showing the five features with the largest F values with binary target variable either normal or GA1. **Abbreviations:** C10—decanoylcarnitine, C12—dodecanoylcarnitine, C14:1—tetradecenoylcarnitine, C18:1—octadecenoylcarnitine, C5—isovalerylcarnitine, C8—octanoylcarnitine, Hci—homocitrulline, Glut—glutarylcarnitine, Glu—glutamic acid.

(A) Full NBS Data Set				(B) Suspected Diagnosis Data Set			
Feature	Normal (μ±σ)	GA1 (μ±σ)	F Value	Feature	Normal (μ±σ)	GA1 (μ±σ)	F Value
Glut	0.2 ± 0.1	2.7 ± 1.5	17,270.68	Glut	0.5 ± 0.1	2.7 ± 1.5	757.7
Hci	1.6 ± 0.8	2.7 ± 1.4	17.4	C10	0.2 ± 0.1	0.1 ± 0	10.1
C5	0.1 ± 0.1	0.2 ± 0.1	13.2	C14:1	0.3 ± 0.1	0.1 ± 0.1	5.7
Glu	400 ± 105	524 ± 108	12.6	C8	0.2 ± 0.1	0.1 ± 0.1	5.6
C18:1	1 ± 0.3	1.2 ± 0.4	10.1	C12	0.3 ± 0.1	0.1 ± 0.1	5.3

**Table 2:** Machine learning classification results for GA1 compared from (**A**) traditional screening results, (**B**) the full data set, (**C**) the suspected diagnosis data set, and (**D**) algorithms optimized for 100% sensitivity on the suspected diagnosis data set. The best-performing features were selected with ANOVA. The methods were evaluated by sensitivity $S_{n}$ and specificity $S_{p}$ calculated from the mean results of ten repeats of 5-fold cross-validation (CV), as well as the number of false positives (FP), false negatives (FN), true negatives (TN), and true positives (TP) (real numbers are rounded up). For (**A**,**B**) the train and validation set consists of 1,025,944 normal and 9 GA1 profiles and the test set consists of 257,414 normal and 2 GA1 profiles. For (**C**,**D**) the train and validation set consists of 485 normal and 9 GA1 profiles and the test set consists of 235 normal and 2 GA1 profiles. For (**C**,**D**), the specificity and sensitivity were calculated based on the full data set to allow comparability between data sets. **Abbreviations:** C10—decanoylcarnitine, C14:1—tetradecenoylcarnitine, Glut—glutarylcarnitine.

Method	Features	Train + Validation Set				CV		Test Set			
		FN	FP	TN	TP	$S_{n}$ (%)	$S_{p}$ (%)	FN	FP	TN	TP
**(A) TRADITIONAL NEWBORN SCREENING**											
NBS	Glut	0	485	1.03 M	9	100	99.9527	0	235	0.26 M	2
**(B) FULL DATA SET**											
LR	Glut, C10	0	31	1.03 M	9	99.11	99.996	0	16	0.26 M	2
RR	Glut, C10, C14:1	3	735	1.03M	6	92.44	99.25	1	71	0.26 M	1
SVM	Glut, C10	0	87	1.03 M	9	92.44	99.999	0	23	0.26 M	2
**(C) SUSPECTED DIAGNOSIS DATA SET**											
LR	Glut, C10	0	24	461	9	86.67	99.999	0	18	217	2
RR	Glut, C10	0	69	416	9	84.67	99.997	0	35	200	2
SVM	Glut, C10	0	33	452	9	90.89	99.998	0	20	215	2
**(D) SUSPECTED DIAGNOSIS DATA SET OPTIMIZED ($100\%\;S_{n}$)**											
LR-100	Glut, C10	0	147	338	9	100	99.989	0	115	120	2
RR-100	Glut, C10	0	235	250	9	100	99.981	0	146	89	2
SVM-100	Glut, C10	0	164	321	9	100	99.987	0	123	112	2

## Data Availability

The NBS data that support the findings of this study are not publicly available due to privacy restrictions. Supplementary data that support the findings of this study are available in the supplementary material of this article (Supplementary Tables S1–S3).

## Supplementary Materials

The following supporting information can be downloaded at: https://www.mdpi.com/article/10.3390/ijns10040083/s1, Figure S1: Data extraction and data cleaning flow chart for newborn screening data; Figure S2: Box plots with distribution of Glut, Hci, and C10 in normal, false positive and GA1 newborn screening profiles; Table S1: Data overview; Table S2: Patient subgroup analysis; Table S3: Feature selection overview.

## Abbreviations

The following abbreviations are used in this manuscript:

3-OH-GA	3-hydroxyglutaric acid
ANOVA	analysis of variance
C14:1	tetradecenoylcarnitine
C5	isovalerylcarnitine
C10	decanoylcarnitine
GA1	glutaric aciduria type 1
Glut	glutarylcarnitine
GDPR	general data protection regulation
Hci	homocitrulline
LR	logistic regression
ML	machine learning
NBS	newborn screening
RR	logistic ridge regression
SVM	support vector machine
UKHD	Heidelberg University Hospital
