# Supplementary material for: Digital-Tier Strategy Improves Newborn Screening for Glutaric Aciduria Type 1
Source: Int J Neonatal Screen. 2024 Dec 21;10(4):83. doi: 10.3390/ijns10040083 (PMC11679506; doi:10.3390/ijns10040083)
Supplement: Supplementary file 1 [file IJNS-10-00083-s001.zip › Supplemental Materials.pdf]

Supplemental Materials: Digital-Tier Strategy Improves Newborn Screening for Glutaric Aciduria Type 1

1. **Supplementary Table** S**1:** Data overview
   - **A:** Overview of all metabolite concentrations and additional variables screened at the NBS laboratory at Heidelberg University Hospital.
   - **B:** Mean and standard deviation of metabolite concentrations of different groups of newborn in newborn screening for GA1 at the NBS laboratory at Heidelberg University Hospital.
2. **Supplementary Table** S**2:** Patient subgroup analysis
   - **A:** Results of further analysis of patient groups with suspected GA1
3. **Supplementary Table** S**3:** Feature selection overview
   - **A:** Results of Wilcoxon rank sum test and ANOVA on full data set (p values and F values) for every feature in the NBS data set.
   - **B:** Results of Wilcoxon rank sum test and ANOVA on suspected diagnosis data set (p values and F values) for every feature in the NBS data set.
4. **Supplementary Figure** S**1:** Data extraction and data cleaning flow chart for newborn screening data.
5. **Supplementary Figure** S**2:** Box plots with distribution of Glut, Hci, and C10 in normal, false positive and GA1 newborn screening profiles.

Supplementary Figures

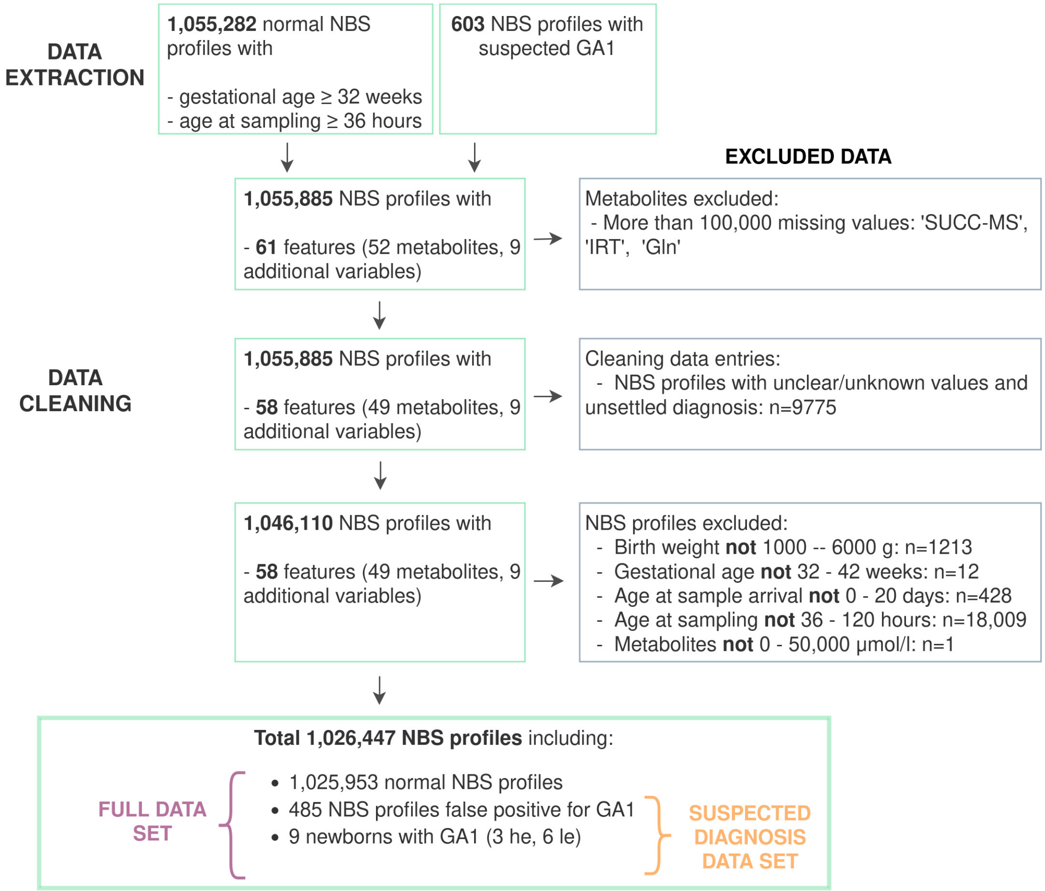

Figure S1: Data extraction and data cleaning flow chart for NBS data screened at the NBS laboratory at Heidelberg University Hospital between 2014 and 2021. NBS profiles from normal and newborns with suspected GA1 are extracted. From both data sets, features and NBS profiles are excluded due to missing entries and implausible values resulting in 1,025,953 normal NBS profiles, 485 false-positive profiles for GA1, and 9 newborns with GA1 including 3 high excretors (he) and 6 low excretors (le). Metabolite abbreviations can be found in Supplementary Table S1 (A).

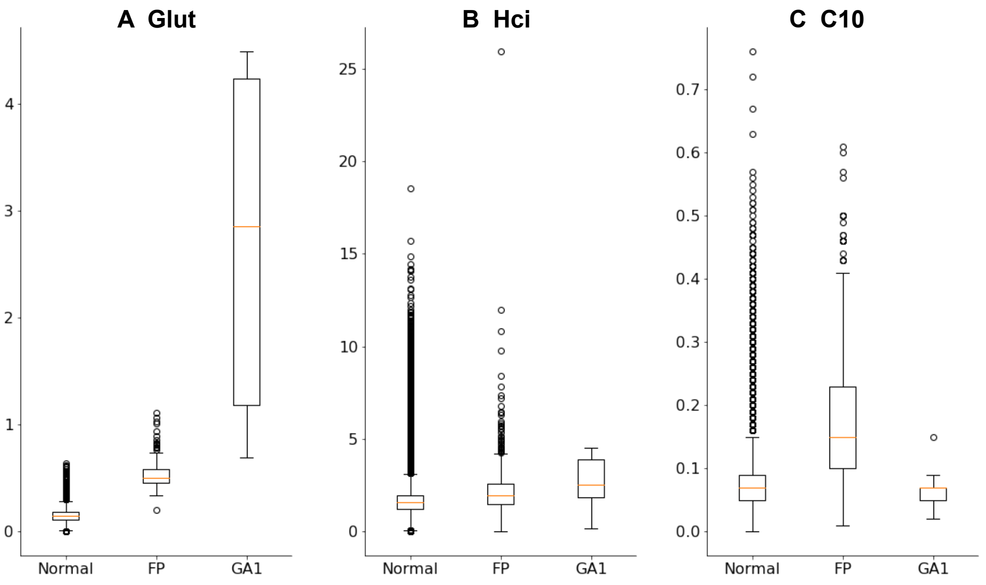

Figure S2: Box plots showing the distribution of the metabolite concentrations of gluatrylcarnitine (Glut), homocitrulline (Hci), and decanoylcarnitine (C10) in newborns with a normal newborn screening profile and newborns with false-positive or GA1 screening results.
